# Novel high reactive bifunctional five- and six-membered bicyclic dicarbonate – synthesis and characterisation†

**DOI:** 10.1039/c8ra00669e

**Published:** 2018-03-27

**Authors:** M. Tryznowski, A. Świderska

**Affiliations:** Warsaw University of Technology, Faculty of Production Engineering Narbutta 85 02-524 Warsaw Poland m.tryznowski@wip.pw.edu.pl; Warsaw University of Technology, Faculty of Chemistry Noakowskiego 3 00-664 Warsaw Poland

## Abstract

Five- and six-membered bicyclic carbonates are valuable raw materials for the synthesis of environmentally friendly polymers, such as polycarbonates or non-isocyanate poly(hydroxyurethane)s. However, bicyclic diglycerol dicarbonates bearing five-membered and six-membered rings have been never reported before. In this work, for the first time, we report a simple procedure for the synthesis of this monomer from commercially available diglycerol. The product was characterised by ^1^H NMR, ^13^C NMR, FTIR spectroscopy and X-ray diffraction measurements. Next, the reactivity of the obtained bicyclic carbonate was investigated. The obtained diglycerol dicarbonate was used as a monomer for polycarbonate and non-isocyanate poly(hydroxyurethane) based on putrescine. In the homopolymerisation reaction the opening of the six-membered carbonate ring was observed, while in the polycondensation with diamine both carbonate rings open nonselectively.

## Abstract 2

The synthesis of novel, bifunctional diglycerol dicarbonate bearing a five-membered and six-membered cyclic carbonate group is presented.

## Graphical abstract

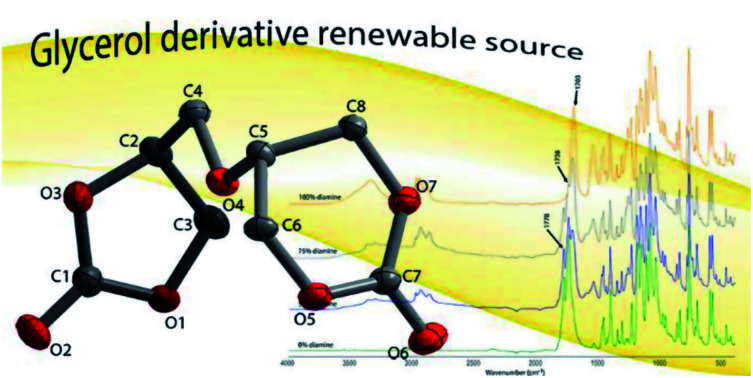

## Introduction

Five- and six-membered bicyclic carbonates are raw materials for the synthesis of polycarbonates or non-isocyanates poly(hydroxyurethane)s *via* environmentally friendly routes. Due to the low thermodynamic stability of six-membered cyclic carbonates, their availability is limited in contrast to five-membered cyclic carbonates.^1^ Furthermore, six-membered cyclic carbonates homopolymerise in contrast to five-membered cyclic carbonates. Finally, the reaction rate constant increases together with the ring size of cyclic carbonates, due to larger ring strain.^2,3^ However, this research focuses on the synthesis and application of five-membered cyclic carbonates, because of the relative ease with which they are synthesised.^4^ Five-membered cyclic carbonates can be prepared from multifunctional oxiranes *via* CO_2_ insertion under mild conditions,^5–7^ transesterification of 1,2-diols with dialkyl carbonates or the reaction of 1,2-halohydrins with bicarbonates.^8–15^ Six-membered cyclic carbonates are obtained by the reaction of oxetanes with CO_2_.^16,17^

Bi- or trifunctional monomers based on carbonates may be used to create multifunctional materials for advanced purposes, for example, in medical science for drug delivery.^18–20^ Bifunctional bicyclic dicarbonate bearing five-membered and six-membered carbonate rings have already been presented in the literature.^21,22^ He *et al.* synthesised in a multistep reaction from trimethylolpropane and used a monomer for poly(hydroxyurethane)s, PHUs, synthesis.^21^ Endo *et al.* presented anionic polymerisation of bifunctional cyclic carbonate consisting of both five- and six-membered rings obtained in a multistep procedure.^22^

Nowadays, the replacement of petroleum based chemicals with renewable raw materials based on biomass is a great challenge for scientists.^23^ Diglycerol is the simplest derivative of cheap glycerol, which is a by-product of biodiesel preparation. However, the worldwide use of glycerol and its derivative is much lower than its production.^24^ So, glycerol and its derivatives may be used as cheap and bio-based raw materials for polycarbonates or (PHUs) obtained by green routes. Diglycerol offered by Solvay S.A. is technical grade (>90%) and contains a mixture of isomers of diglycerol: α,α-diglycerol, α,β-diglycerol, β,β-diglycerol and cyclic diglycerols. In our previous work we demonstrated a one-step procedure for the synthesis of five-membered bicyclic diglycerol dicarbonate as a raw material for PHUs.^8^ Due to the increasing environmental concerns linked with the stringent regulations of the European Union to ensure a high level of protection of health and the environment and the limitations in the use of isocyanates as a raw materials for conventional polyurethanes synthesis, PHU based on fossil or bio-based raw materials have been gain on importance.^25–28^

**Scheme 1:**
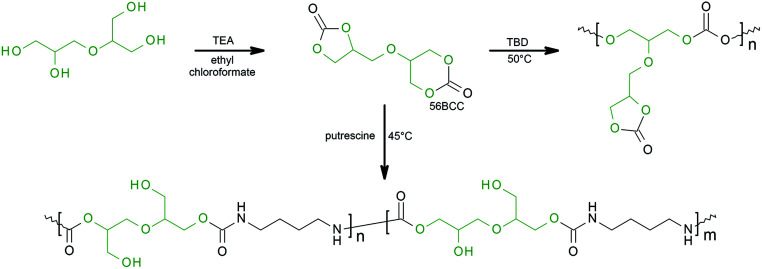
Synthesis of α,β-diglycerol dicarbonate.

For the first time, we report a one-step procedure for the synthesis of novel and unknown derivatives of diglycerol, bifunctional bicyclic dicarbonate bearing five-membered and six-membered functional groups, α,β-diglycerol dicarbonate (56BCC). The described procedure allows the synthesis of bicyclic dicarbonate with good yields from an easily available starting material, commercially available diglycerol. The product was characterised by FTIR spectroscopy, NMR techniques (^1^H NMR, ^13^C NMR, ^1^H–^1^H COSY and ^1^H–^13^C HSQC) and X-ray diffraction measurements. Furthermore, the reactivity of the obtained α,β-diglycerol dicarbonate is also discussed. The obtained α,β-diglycerol dicarbonate was used as a monomer for polycarbonate and poly(hydroxyurethane)s base on putrescine (see Scheme 1).

## Experimental

### Materials

Diglycerol (α,α-diglycerol ∼84%, α,β-diglycerol ∼14%, β,β-diglycerol, <1%, cyclic diglycerols ∼0.2%) was a gift from Solvay S.A. (Brussels, Belgium). All of the reagents were purchased from Sigma-Aldrich (Poznań, Poland) and used as received, without further purification.

### Instrumentation

FTIR spectra were recorded on a Biorad FTS-165 FTIR spectrometer using KBr pellets or a Bruker Alpha FTIR spectrometer equipped with a Platinum ATR single reflection diamond ATR module. ^1^H and ^13^C NMR spectra were recorded on a Varian VXR 400 MHz spectrometer using tetramethylsilane as an internal standard and deuterated solvents (DMSO-d6) and analysed with MestReNova v.11.0.4 (Mestrelab Research S.L.) software. Two-dimensional analyses comprising ^1^H–^1^H COSY (correlation spectroscopy), ^1^H–^13^C HMBC (heteronuclear multi-bond correlation spectroscopy) and ^1^H–^13^C HSQC (heteronuclear single-quantum correlation spectroscopy) were also performed.

The X-ray measurements were performed at 100(2) K on a Bruker D8 Venture Photon100 CMOS diffractometer equipped with a TRIUMPH monochromator and a MoKα fine focus sealed tube (*λ* = 0.71073 Å). A total of 5623 frames were collected with the Bruker APEX2 program.^29^ The frames were integrated with the Bruker SAINT software package^30^ using a narrow-frame algorithm. The data were corrected for absorption effects using the multi-scan method (SADABS).^31^ All non-hydrogen atoms of the fragment with higher occupancy were refined anisotropically, while all hydrogen atoms were placed in calculated positions and refined within the riding model. The atomic scattering factors were taken from the International Tables.^32^ Molecular graphics were prepared using the program Diamond 3.2.^33^ Thermal ellipsoids parameters are presented at 50% probability level.

MALDI-TOF measurements were performed on a Bruker UltraFlex MALDI TOF/TOF spectrometer (Bremen, Germany) in a linear or reflectron mode using a DHB (2,5-dihydroxybenzoic acid) or HABA 2-(4′-hydroxybenzeneazo) benzoic acid matrix and Bruker Peptide Calibration Standard (1047.19 × 10^3149.57^ Da) as a calibrant and analysed with flexAnalysis v.3.3 (Bruker Daltonik Gm) and Polymerix v. 2.0 (Sierra Analytics Inc.) software.

### Synthesis of α,β-diglycerol dicarbonate (56BCC)

11.58 g (69.8 mmol) of the tetrol mixture, obtained as a residue of a reaction described in our previous work,^8^ was placed in a 500 ml three neck round bottom flask equipped with a mechanical stirrer, a thermometer and a nitrogen inlet, followed by 250 ml THF. Then, the mixture was stirred for 30 min at 50 °C in order to dissolve the tetrols mixture. The reaction mixture was cooled down to 0 °C and 30.39 g (0.28 mol) ethyl chloroformate was added. Then, 28.33 g (0.28 mol) of triethylamine was added dropwise. The reaction mixture was stirred under an inert gas atmosphere at 0 °C for 24 h and was gradually warmed up to room temperature. Next, the reaction mixture was filtered under reduced pressure and the filtrate was left to crystallise at −5 °C. The yield of the reaction was 39% (4.52 g).

FTIR: (cm^−1^): 2968, 1775, 1737, 1402, 1229, 1185, 1158, 1112, 1081, 1037, 767, 703, 601, 279; ^1^H NMR (400 MHz, DMSO-*d*_6_): *δ* (ppm) = 4.99–4.92 (m, 1H, CHOC(O)), 4.58–4.48 (m, 3H, OCH_2_), 4.43–4.34 (m, 2H, CH_2_O), 4.22 (dd, 1H, *J* = 8.4, 6.1 Hz), 4.01 (p, 1H, *J* = 2.0 Hz, 1H), 3.85–3.72 (m, 2H); ^13^C NMR (DMSO-d_6_, 100 MHz); 154.9, 147.4, 75.3, 69.6, 69.4, 67.5, 67.2, 66.0.

### Homopolymerisation of 56BCC

0.42 g (5 mmol) of 56BCC was dissolved in 9 ml of acetonitrile in a 25 ml round bottom flask equipped with an argon inlet. Next, 1 mg (10 mmol) of TBD was added. The polymerisation was conducted under an inert gas atmosphere at 50 °C for 32 h. Then, the acetonitrile was evaporated. The polymer was obtained as a transparent, viscous liquid.

FTIR: (cm^−1^) = 3729, 2958, 2913, 2875, 1790, 1746, 1518, 1453, 1241, 1239, 1173, 1109, 1079, 951, 913, 845, 772, 710, 714, 619; ^1^H NMR (400 MHz, DMSO-d_6_): *δ* (ppm) = 4.98–4.81 (m, 1H, OCH), 4.59–4.43 (m, 1.6H, OCH_2_), 4.31–3.85 (m, 5.9H, OCH_2_, OCH), 3.84–3.63 (m, 2.1H, CHCH_2_O); ^13^C NMR (DMSO-d_6_, 100 MHz); 154.91, 154.34, 75.45, 70.40, 69.11, 66.24, 65.80.

### Synthesis of poly(hydroxyurethane)

0.2 g (91 mmol) of 56BCC was dissolved in DMSO-d_6_ in a 10 ml vial. Next 0.5 g (91 mmol) of 1,4-diaminobutane was added dropwise. The polymerisation was conducted under an inert gas atmosphere (argon) at 45 °C for 72 h.

FTIR: (cm^−1^) = 3290, 2929, 1703, 1536, 1402, 1230, 1156, 1079, 1038, 783, 601; 1H NMR (DMSO-d_6_, 400 MHz); *δ* (ppm) = 7.28 (m, 1.62H, NH_(E)_), 6.85 (bs, 0.19H, NH_(Z)_), 4.90 (bs, 0.29H, OH_secondary_), 4.79 (bs, 1.13H, OH_primary_), 4.56–4,46 (m, 1.67H, OCHCH_2_OH), 4.33–4.20 (m, 0.91H, OCHCH_2_OH), 4.14–4.00 (m, 1.8H, CHCH_2_O), 3.94–3.76 (m, 2.76H, CH_2_CHCH_2_), 3.74–3.63 (m, 0.9H, CHOH), 3.56–3.38 (m, 3.6H, CH_2_OH), 3.00 (bs, 3.81H, CH_2_NH), 1.45–1.20 (bs, 4.06H, CH_2_ CH_2_); ^13^C NMR (DMSO-d_6_, 100 MHz); 156.17, 155.05, 79.5, 75.69, 75.66, 68.97, 66.04, 66.01, 63.20, 60.44, 60.37, 41.20, 30.58, 26.99.

## Results and discussion

### Synthesis of α,β-diglycerol dicarbonate

As a starting material for the synthesis of α,β-diglycerol decarbonate, 56BCC, commercially available diglycerol (Solvay) was used (Scheme 1). Diglycerol is a mixture of various isomers α,α-, α,β- and β,β-diglycerol and cyclic diglycerols. The reaction was performed in two steps. For the first step, the α,α-diglycerol isomer was quantitatively separated. In our previous work we presented a simple, one-step synthesis of five-membered diglycerol dicarbonate from the α,α-diglycerol isomer.^8^ For the second step, the reaction residue may be used as a substrate for the synthesis of the five- and six-membered diglycerol dicarbonate. The reaction of α,β- and β,β-diglycerol and cyclic diglycerols in the presence of ethyl chloroformate leads to bicyclic carbonate. However, the 1,2-diol group transforms into a five-membered carbonate ring while the 1,3-diol group transforms into a six-membered carbonate ring. It should be highlighted, that for the first time the derivative of diglycerol dicarbonate bearing five-membered and six-membered carbonate groups occur simultaneously. We suppose that at the third step, repeating the synthesis strategy described in this work and previous work,^8^ bicyclic dicarbonate of β,β-diglycerol may be obtained.

The structure of the obtained product was confirmed by FT-IR and several NMR techniques (^1^H NMR, ^13^C NMR, ^1^H–^1^H COSY and ^1^H–^13^C HQSC).

**Fig. 1:**
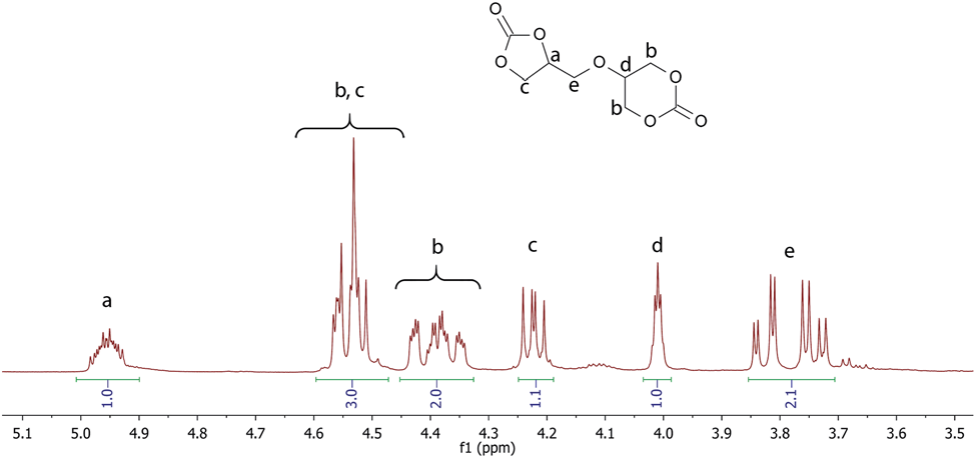
^1^H NMR of 56BCC.

The formation of the bicyclic dicarbonate was followed by FTIR spectroscopy. Fig. 1 shows the FT-IR spectrum of the final product. The characteristic absorption band corresponding to the five-membered and six-membered cyclic carbonate carbonyl group appears at 1798 cm^−1^ and 1747 cm^−1^, respectively. The absorption band for the hydroxyl group is not observed.

The ^1^H NMR spectrum of 56BCC is shown in Fig. 1. The proton of the chiral carbon (a) of the obtained diglycerol dicarbonate appears as a multiplet at approximately 4.95 ppm. The methylene protons of the five-membered cyclic ring (c) appear as two pairs of doublets at approximately 4.53 ppm and 4.22 ppm. However, the protons (c) are covered by the methylene group protons of the six-membered cyclic ring (b). The methylene group (e) appears as a doublet of doublet of doublet in the range of 3.85–3.71 ppm.

**Fig. 2:**
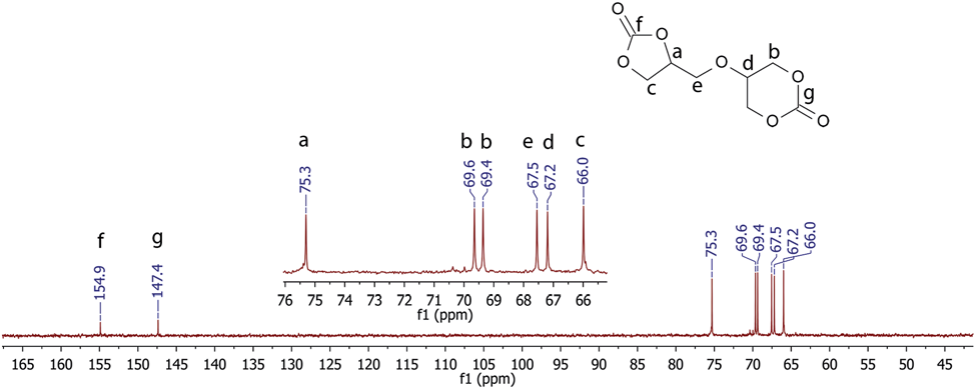
^13^C NMR of 56BCC.

The ^13^C NMR spectrum confirmed the structure of the obtained diglycerol dicarbonate (see Fig. 2.). The carbonyl group carbon in the five-membered and six-membered cyclic carbonate rings appears at 156 and 147 ppm, respectively. Thanks to 2D COSY (Fig. 1S in ESI†), HMBC (Fig. 2S in ESI†) and HSQC (Fig. 3S in ESI†) the structure of the obtained 5CC-6CC diglycerol dicarbonate was corroborated.

**Fig. 3:**
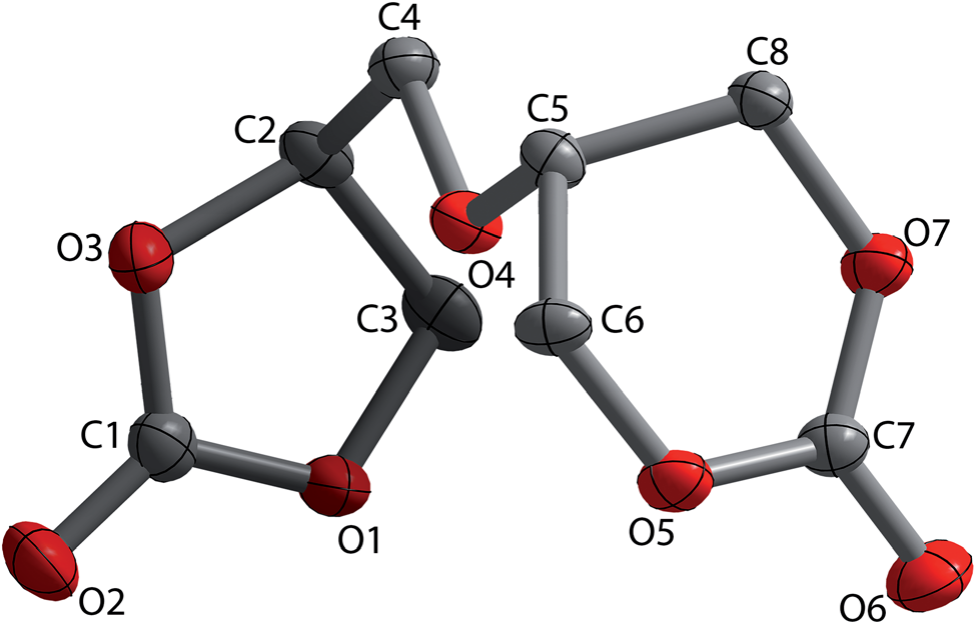
X-ray structure of 56BCC.

The structure of the 56BCC product was determined by single crystal X-ray diffraction. Single crystals of α,β-diglycerol dicarbonate were obtained by recrystallisation from an acetonitrile–ethyl acetate solution of the product. Fig. 3 shows the X-ray crystal structure of the 56BCC. The crystallographic details can be found in the ESI.† α,β-diglycerol dicarbonate was crystallised in a monoclinic crystal system with the space symmetry *P*2_1_/*c*, so there are two separate types of molecules of the same geometry. The r.m.s. deviation was equal to 0.039 Å. The bond lengths C=O in the 6CC group varied by 1.198(5) Å and in 5CC group varied from 1.198(3) to 1.25(2) Å. These values are comparable to those observed for trimethylene carbonate or ethylene carbonate. Furthermore, the bond length of C=O in the 5CC group of diglycerol dicarbonate varied from 1.1947(18) to 1.1968(18) Å.^8^

### Synthesis of polycarbonate

The synthesis of polycarbonate was performed in acetonitrile using TBD as a catalyst. The progress of the reaction was monitored by FT-IR and ^1^H NMR spectroscopy. During the reaction, a characteristic absorption band corresponding to a six-membered cyclic carbonate carbonyl group at 1740 cm^−1^ disappears and a new peak appears at 1626 cm^−1^ corresponding to carbonyl groups of linear carbonate. Interestingly, the ring-opening polymerisation of the five-membered cyclic carbonate group was not observed.

**Fig. 4:**
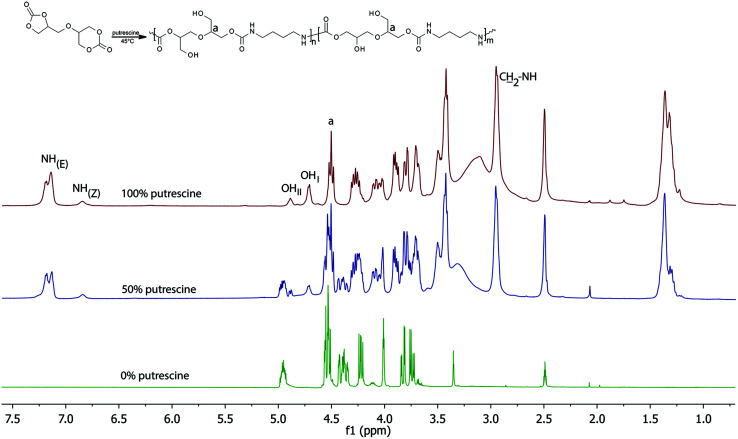
^1^H NMR of PHU formation.

Fig. 4S and 5S in the ESI† show the ^1^H NMR and ^13^C NMR spectra after the polymerisation of 56BCC. The ^1^H NMR and ^13^C NMR spectra confirmed the structure of the product. In the ^1^H NMR, the disappearance of peaks characteristic of six-membered cyclic carbonates protons in methane and methylene groups and the appearance of new peaks characteristic for linear carbonate was observed. At the same time on the ^13^C NMR spectrum, characteristic peaks (at 147.4, 69.7, 69.3 and 67.2 ppm) for six-membered cyclic carbonate disappears and new peaks (at 154.1, 75.3 ppm) characteristic for linear carbonate appear.

Fig. 6S in ESI† shows the MALDI-TOF mass spectrum of polycarbonate. The mass increment of the repeating unit is 218 Da, and corresponds to a repeating unit of polycarbonate.

### Synthesis of PHUs

The synthesis of 56BCC leading to PHU was performed using putrescine and DMSO-d_6_ as the solvent. The reaction was monitored by FT-IR and ^1^H NMR spectroscopy. Fig. 4 shows the changes to the ^1^H NMR spectra during the reaction. Considering the carbonate ring reactivity, it can be expected, that the condensation reaction of bicyclic carbonate 56BCC should be selective: for the first step, the six-membered carbonate ring should react and then the five-membered carbonate ring should react. However, despite the different reactivity of the five- and six-membered cyclic carbonates groups, the reaction of diglycerol dicarbonate is not selective and both groups react simultaneously. During the reaction, the characteristic peaks corresponding to methine group in five-membered cyclic carbonate group at 4.99–4.92 ppm disappears together with the characteristic peaks corresponding to methylene groups in six-membered cyclic carbonate at 4.01 ppm.

## Conclusions

This study presents for the first time the synthesis of an unknown derivative of diglycerol, a bicyclic diglycerol dicarbonate bearing five-membered and six-membered cyclic carbonate groups. The obtained product was comprehensively characterised FT-IR, ^1^H NMR, ^13^C NMR. Furthermore, the X-ray structure of α,β-diglycerol dicarbonate, 56BCC, showed that the obtained bis(cyclic carbonate) was a pair of enantiomers.

The reactivity of α,β-diglycerol dicarbonate was investigated. The 56BCC was successfully used as a monomer for the synthesis of polycarbonate and poly(hydroxyurethane). Furthermore, in the homopolymerisation reaction the opening of the six-membered carbonate ring was observed, while in the polycondensation with putrescine leading to PHU, both carbonate rings open nonselectively.

## Conflicts of interest

There are no conflicts to declare.

## Supplementary Material

RA-008-C8RA00669E-s001

RA-008-C8RA00669E-s002
