# Supplementary material for: Novel high reactive bifunctional five- and six-membered bicyclic dicarbonate – synthesis and characterisation
Source: RSC Adv. 2018 Mar 27;8(21):11749–53. doi: 10.1039/c8ra00669e (PMC9079124; doi:10.1039/c8ra00669e)
Supplement: RA-008-C8RA00669E-s001 [file RA-008-C8RA00669E-s001.pdf]

Electronic Supplementary Material (ESI) for RSC Advances.
This journal is © The Royal Society of Chemistry 2018

ELECTRONICAL SUPLEMENTARY MATERIALS

for

Novel high reactive bifunctional five- and six-membered bicyclic dicarbonate – synthesis and characterisation

Mariusz Tryznowski[a,] * and Aleksandra Świderska[b]

[a] Faculty of Production Engineering, Warsaw Univeristy of Technology, Narbutta 85, 02-524 Warsaw, Poland

[b] Faculty of Chemistry, Warsaw University of Technology, Noakowskiego 3, 00-664 Warsaw, Poland

*e-mail: m.tryznowski@wip.pw.edu.pl

Table of contents:

Table 1S. Crystal data and structure refinement for *α,β*-diglycerol dicarbonate (56BCC)

Figure 1S ^{1}H–^{1}H COSY of 56BCC

Figure 2S ^{1}H–^{13}C HMBC of 56BCC

Figure 3S ^{1}H–^{13}C HQSC of 56BCC

Figure 4S ^{1}H NMR of polycarbonate

Figure 5S ^{13}C NMR of polycarbonate

Figure 6S Maldi-ToF of polycarbonate

Figure 7S FT-IR of PHU formation

Table 1S. Crystal data and structure refinement for *α,β*-diglycerol dicarbonate, 56BCC

Identification code	MTAS_MON1	
Chemical formula	$C_8H_{10}O_7$	
Formula weight	218.16	
Temperature	100(2) K	
Wavelength	1.54178 Å	
Crystal size	0.199 x 0.281 x 0.298 mm	
Crystal habit	colorless prism	
Crystal system	Monoclinic	
Space group	P1 21/c 1	
Unit cell dimensions	a = 10.5437(12) Å	α = 90°
	b = 8.0245(9) Å	β = 93.598(2)°
	c = 10.8487(12) Å	γ = 90°
Volume	916.08(18) Å^3	
Z	4	
Density (calculated)	1.582 g/cm^3	
Absorption coefficient	1.246 mm^{-1}	
F(000)	456	

Diffractometer	Bruker D8 VENTURE PHOTON 100 CMOS
Radiation source	INCOATEC IμS micro-focus source, CuKα
Theta range for data collection	6.87 to 67.46 deg
Index ranges	-12<=h<=12, -9<=k<=9, -12<=l<=12
Reflections collected / unique	10146 / 1641 [R(int) = 0.0251]
Completeness to theta = 28.00	99.6%
Absorption correction	Multi-scan
Max. and min. transmission	0.7900 and 0.7080
Refinement method	Full-matrix least-squares on F^2
Data / restraints / parameters	1641 / 49 / 194
Goodness-of-fit on F^2	1.193
Final R indices [I>2sigma(I)]	R1 = 0.0332, wR2 = 0.0844
R indices (all data)	R1 = 0.0340, wR2 = 0.0851
Largest diff. peak and hole	0.284 and -0.182 eÅ^{-3}
R.M.S. deviation from mean	0.039 eÅ^{-3}

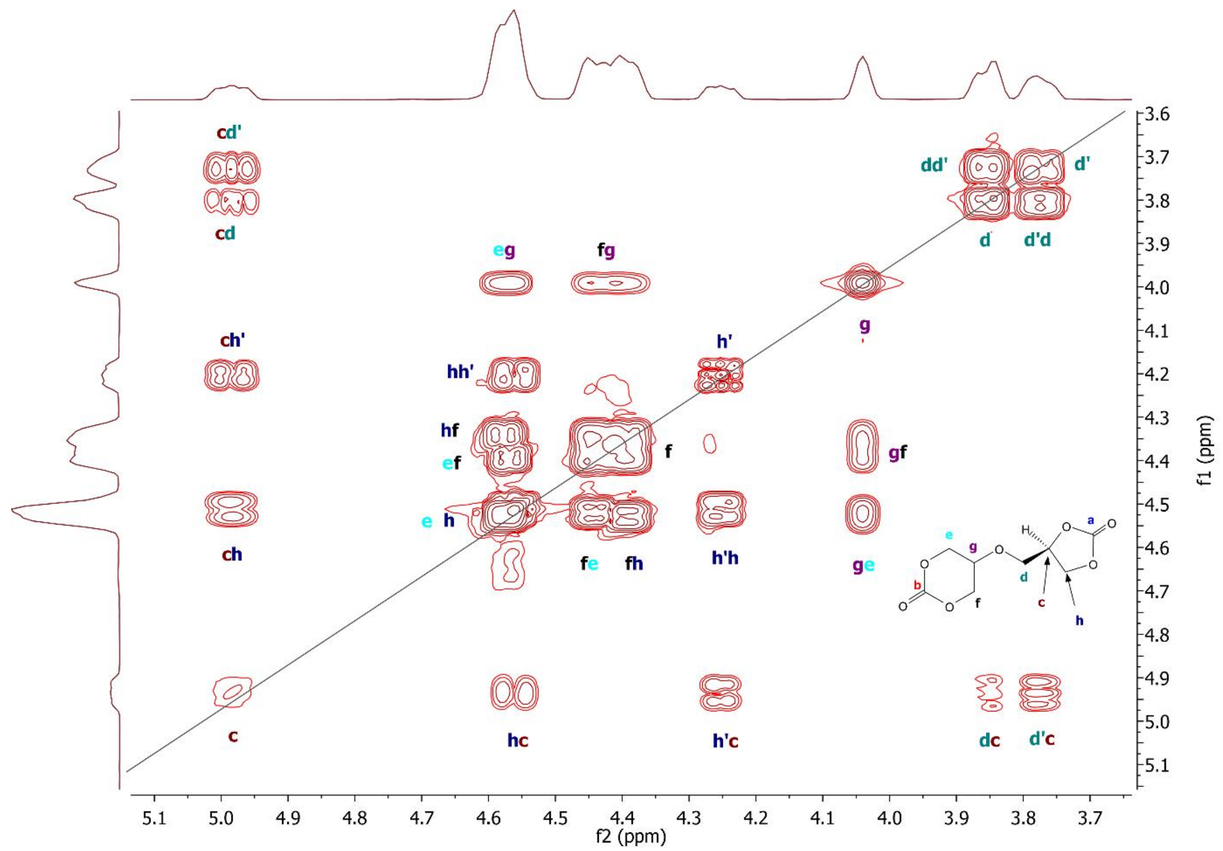

Figure 1S ^{1}H–^{1}H COSY of 56BCC

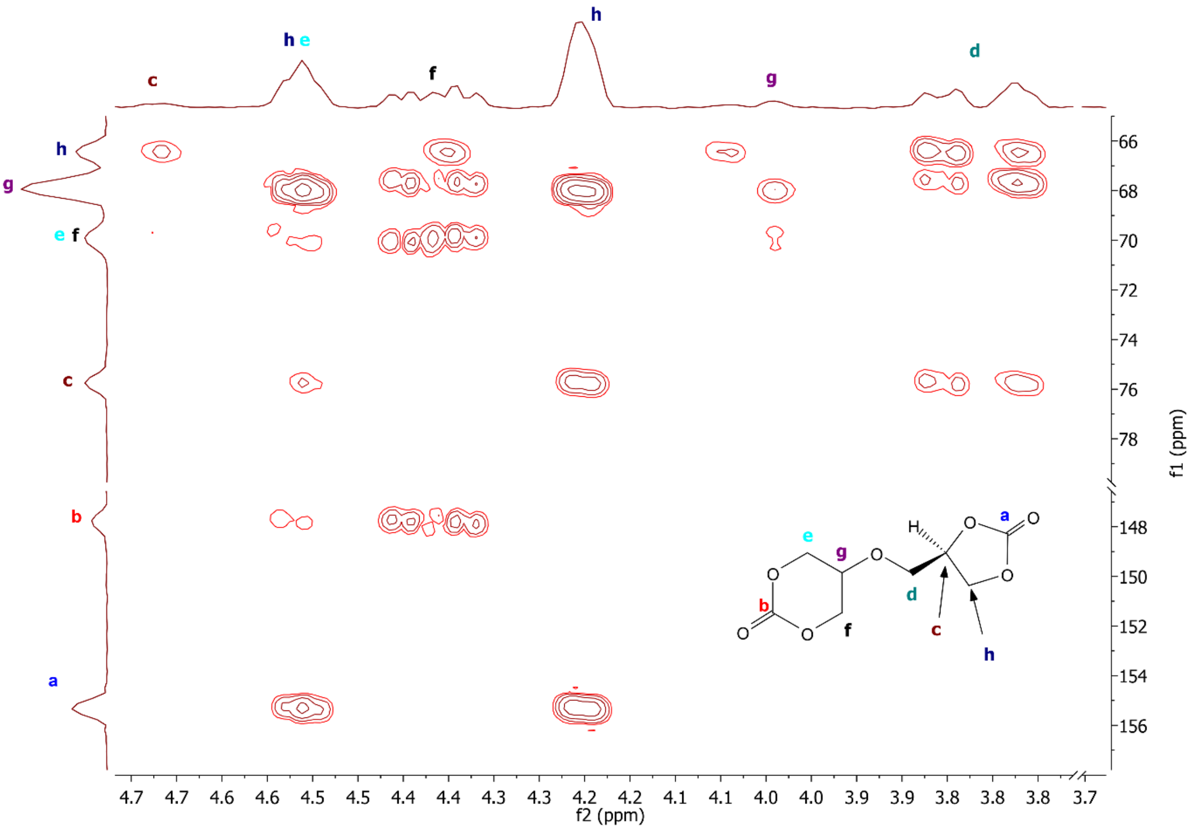

Figure 2S ^1H–^{13}C HMBC of 56BCC

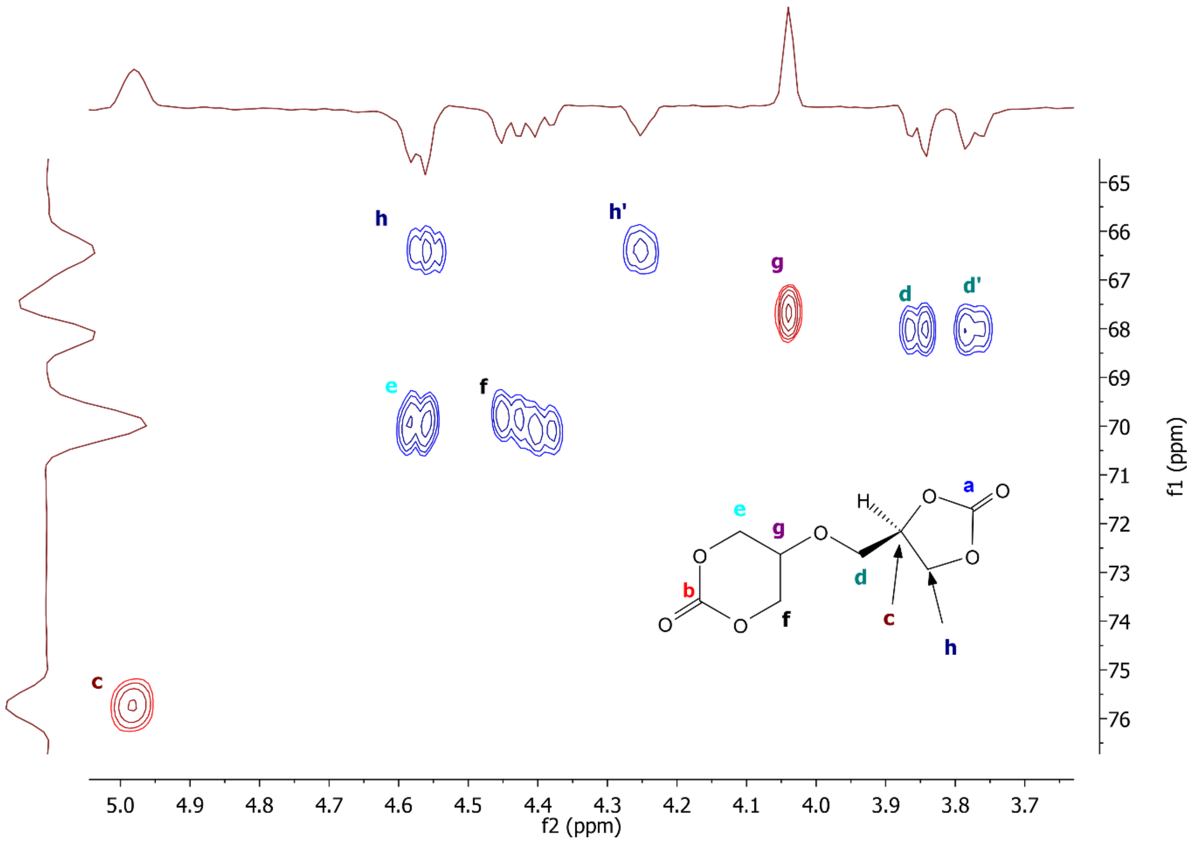

Figure 3S ^{1}H–^{13}C HQSC of 56BCC

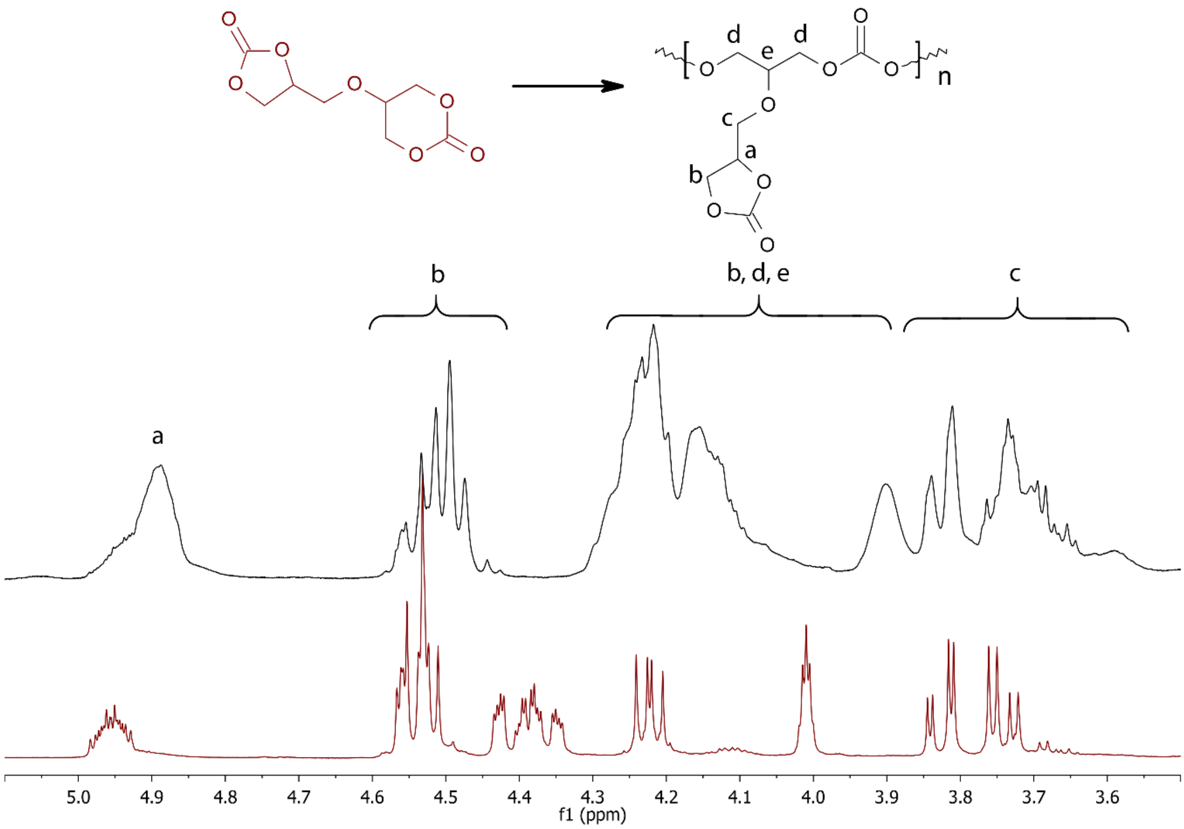

Figure 4S ^{1}H NMR of polycarbonate

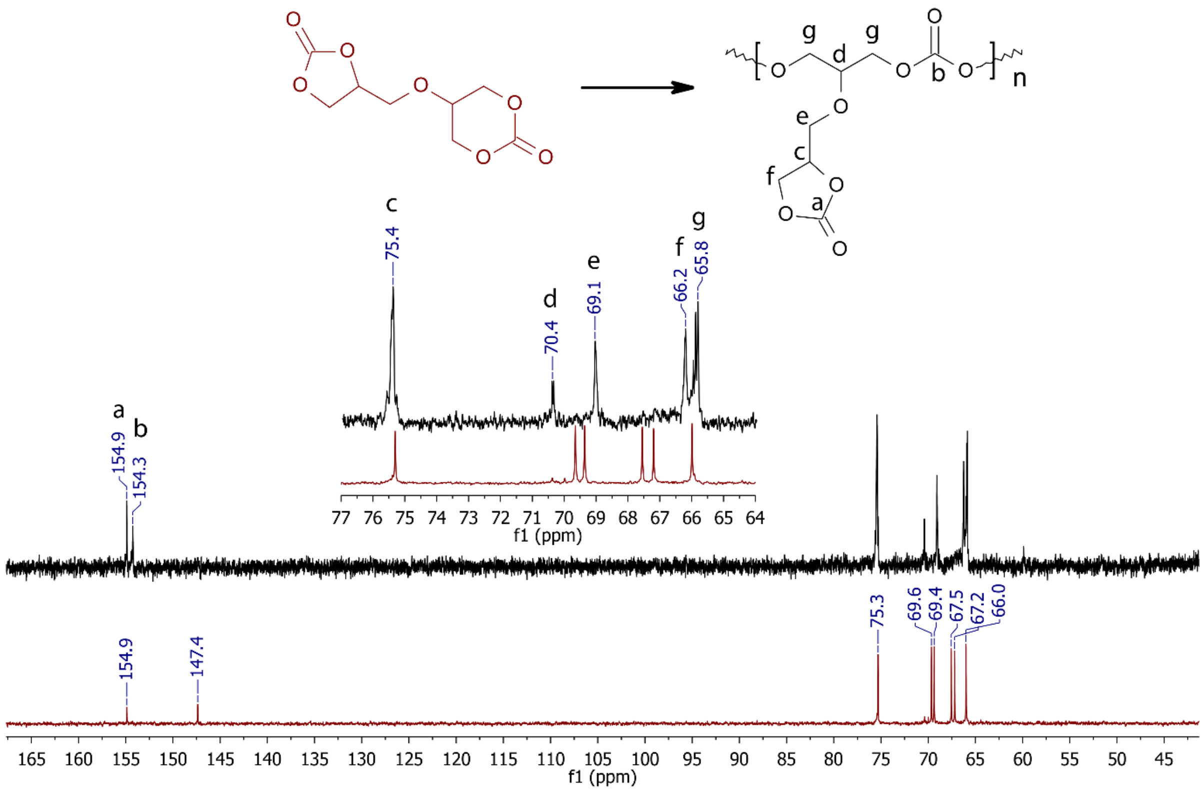

Figure 5S ^{13}C NMR of polycarbonate

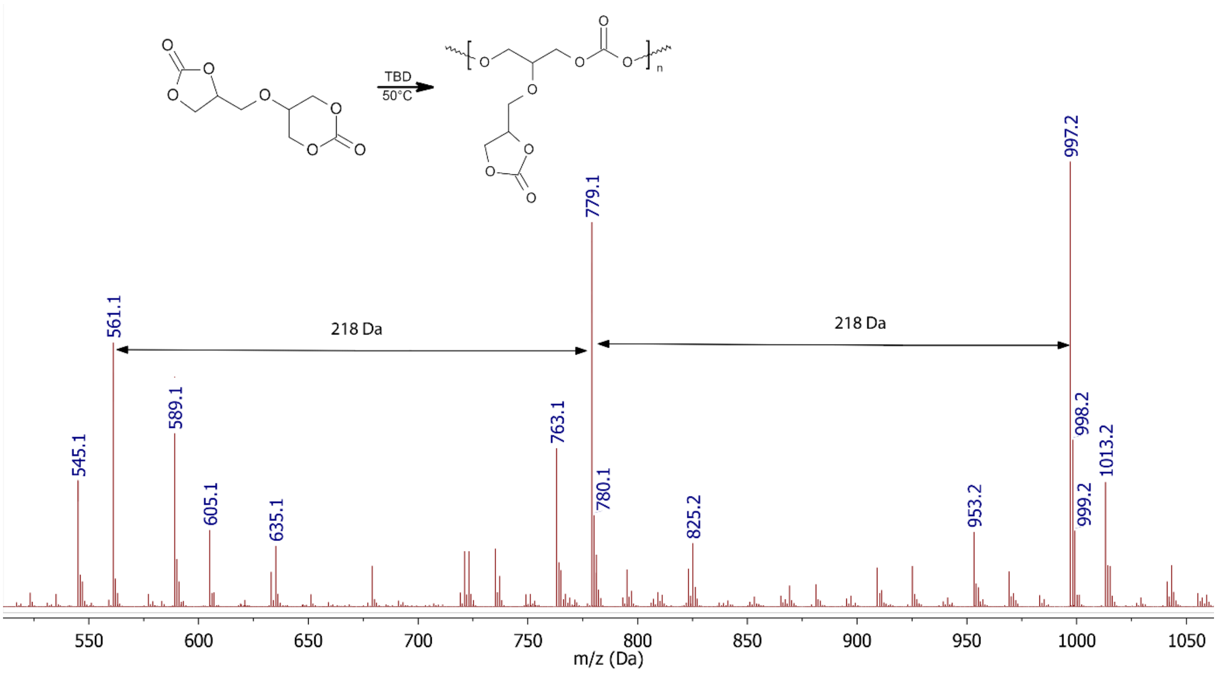

Figure 6S Maldi-ToF of polycarbonate

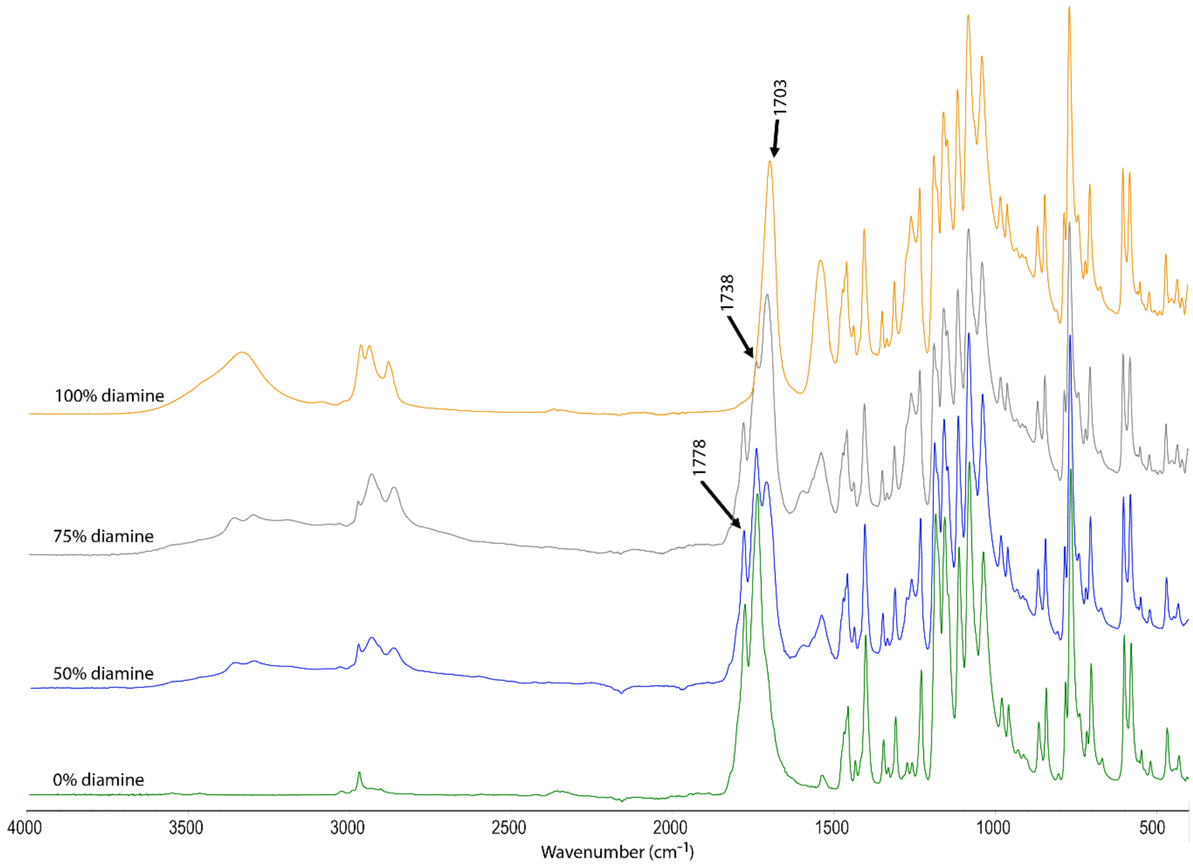

Figure 7S FT-IR of poly(hydroxyurethane)s formation
